# Influence of the use of remediated soil and agricultural drainage water on the safety of tomato fruits

**DOI:** 10.1007/s11356-024-33187-z

**Published:** 2024-04-18

**Authors:** Salah H. Salem, Mohamed Saber, Samir Gadow, Hoda Kabary, Alaa Zaghloul

**Affiliations:** 1 Food Toxicology and Contaminants Dept., Food Industries and Nutrition Institute, National Research Centre, Cairo, 12622 Egypt; 2 Agricultural Microbiology Dept., Agricultural and Biological Sciences Institute National Research Centre, Cairo, 12622 Egypt; 3 Soils and Water Use Dept., Agricultural and Biological Sciences Institute National Research Centre, Cairo, 12622 Egypt

**Keywords:** Tomato, Food safety, DHS, Toxic elements, Soil remediation, Drainage water remediation, Clay minerals, Sustainable agriculture, Soil remediation

## Abstract

The objective of this study is to assess the effectiveness of different techniques employed in remediating contaminated soil and wastewater ecosystems to ensure the safety of tomato fruits (*Solanum lycopersicum* L. var. *cerasiforme*) cultivated in these environments. Three biochemical techniques T1–T3, besides two controls CCU and CCT, were used to remediate contaminated soil ecosystems using rock phosphate, elemental sulfur, bentonite, phosphate-dissolving bacteria, and *Thiobacillus* sp. The contaminated agricultural drainage water was remediated by a down-flow hanging sponge (DHS) system. Two experiments were conducted: a pot experiment took place in the greenhouse at the National Research Center of Cairo (Egypt) and a field experiment was carried out at the basin site in the village of El-Rahawy, applying the optimal treatment(s) identified from the greenhouse experiment. The health risk assessment for potentially toxic elements (PTEs) in the harvested tomato fruits was conducted by calculating estimated daily intake (EDI) and target risk quotient (THQ) values. Results from the greenhouse experiment indicated the high effectiveness of the DHS technique in remediating El-Rahawy agricultural drainage water. The content of PTEs after remediation was significantly reduced by 100%, 93.3%, 97.8, and 77.8% for cadmium, copper, manganese, and zinc, respectively. The application of treated drainage water in employed reclaimed soil ecosystems led to a remarkable decrease in PTE levels, especially under T3 treatment; the reduction reached 89.4%, 89.5%, and 78.4% for nickel, copper, and zinc, respectively. The bioremediation technique also reduced the content of PTEs in tomato fruits harvested from both greenhouse and field experiments; the cadmium content, for example, was below detection limits in all treatments. The T3 treatment applied in the greenhouse experiment caused the highest percentage decrease among the employed PTEs in tomato fruits grown in the greenhouse. The same trend was also reached in the field experiment. Microbiological analyses of tomato fruits revealed that *E*. *coli*, *Salmonella*, or *S. aureus* bacteria were identified on tomato fruits harvested from either greenhouses or field experiments, showing that the counted total bacteria were higher under the field experiment compared to the greenhouse experiment. The health risk assessment parameter THQ was below 1.0 for all tested metals under all treatments. This means that no potential health risk is expected from consuming tomato products produced under the different employed remediation treatments. In conclusion, the employed bioremediation techniques successfully reduced the PTE content and microbial load in both soil and drainage water ecosystems and in harvested tomato fruits. Henceforth, no health risks are expected from the consumption of this product.

## Introduction

Recently, food safety measures have become a major concern for public health authorities and organizations. Currently, the perception of risk is more pronounced for biological contaminants than for physical or chemical ones (Rovira et al. 2006). The contamination of soil ecosystems with potentially toxic elements is now regarded as one of the most serious environmental problems facing Egypt. In the country, numerous sources of potentially toxic elements (PTEs) contribute to the contamination of agricultural ecosystems, stemming from industrial and mining activities, metalliferous ores, electroplating factories, and excessive use of fertilizers and pesticides, as well as the discharge of municipal waste, all of which pose serious negative health impacts. PTEs can easily enter the food chain when agricultural soils contaminated with PTEs are used to cultivate food and forage crops (Abdel-Rahman 2022; Abdel-Rahman et al. 2022; Wang et al. 2003). Tomatoes (*Solanum lycopersicum* L. var. *cerasiforme*) are an essential component of the Egyptian human diet, by eating it directly in salads or using it in cooking. El-Azeem Ahmed et al. (2023) investigated the effects of PTEs on the growth characteristics of produced tomatoes grown under wastewater irrigation; they concluded that the nutrients (N, P, and K) inside cultivated tomato plants were decreased, while PTEs were increased as a result of using wastewater in irrigation. In addition, Enamorado et al. (2014) mentioned evidence of significant enrichment of PTEs such as 226 Ra in the surface horizon of reclaimed soil plots compared to deeper horizons. This enrichment was observed alongside higher concentrations of Cd in tomatoes produced in areas with elevated PTE concentrations compared to those from other regions in Spain (Enamorado Báez et al. 2009; Abril et al. 2008). However, the most severe source of contamination has become the use of contaminated agricultural drainage water for irrigation (Shehata et al. 2019; Salem et al. 2021). There is no doubt that vast areas of vegetable crops are now irrigated with raw agricultural drainage water in Egypt; such a practice led to the harvest of vegetable fruits being chemically and microbiologically contaminated, which for sure harms consumers’ health. In most cases, these types of harvested crops do not meet the required food standards set in the exporting countries, which leads to their rejection and adverse impacts on the national economy (Hoffmann and Vossenaar 2007). Mishra et al. (2023) mentioned that the use of sewage effluents in the irrigation of tomatoes is commonly used under freshwater scarcity. They discovered that these types of low-quality waters include many *E. coli* and fecal enterococci over the reuse limit (Libutti et al. 2018). Researchers also reported that the application of low-quality water in irrigation gets a concentration of *E. coli* above the threshold limit of Italian law (Gatta et al. 2016). Mcheik et al. (2017) discovered higher quantities of fecal coliforms than the WHO-suggested limit of 1000 CFU/100 ml in an experimental study in Lebanon.

Minimizing the hazards of both organic and inorganic pollutants in soils and low-quality waters used for irrigation was reported by different authors. Bolan et al. (2014) mentioned that the availability of certain PTEs could be decreased or eliminated by the addition of sorbent/precipitating amendments to the aqueous soil solution. In other words, immobilizing agents could be used to decrease metals or metalloid invasion into the food chain through plant uptake. According to Essa and Farragallah (2006), clay or modified clay minerals are necessary and low-cost materials to prevent contaminants from migrating from the soil ecosystem and the surrounding environment. They were among the main components that interact with almost all soil contaminants. Because of their ability to retain both organic and inorganic pollutants, clays and modified clays frequently serve as a short-term sink for PTEs in soil ecosystems. Asaad et al. (2013) mentioned that both raw and modified clay minerals were long-established as successful adsorbents for the elimination of hazardous metal/metalloid toxic ions that represent actual concern to the nearby ecosystem. Montmorillonite was reported to show the best suitability to adsorb and retain the most toxic PTEs such as Zn, Cd, Cr, Co, Pb, Cu, Mn, Ni, and Fe from the soil ecosystem (Satje and Nelson 2009).

The food-borne illness outbreaks linked with fresh produce consumption have increased due to changes in personal consumption, greater availability of produce worldwide, and increased numbers of immune-compromised consumers (Beuchat 2002; Warriner et al. 2009). There is no doubt that *E. coli* and *Salmonella* are the major pathogens that cause food-borne outbreaks associated with the ingesting of fresh vegetables and crops (FDA 1998; Buck et al. 2003; Warriner et al. 2009). Microbial contamination of fresh food products could occur at any stage, from the farmer to the consumer, beginning with production, harvest, processing, storage, transport, retailing, and handling by the consumer at home (FDA 2001; WHO/FAO 2008).

The International Conference on Food Safety, which took place in Addis Ababa in February 2019, and the International Forum on Food Safety and Trade, which took place in Geneva the following year, both defended the role of food safety in achieving sustainable development goals. Globally, unsafe vegetables and fruits pose significant health intimidation to infants, toddlers, pregnant women, disabled and elderly people, and those with an undermined health condition. At the time, WHO, in association with FAO, OIE, and other international organizations, ensured the significance of food safety along the entire food chain, starting from production to consumption. One of the agricultural techniques to improve yield and crop safety could be growing plants in a remediated soil ecosystem irrigated with remediated low-quality water, e.g., agricultural drainage water. The most critical food safety indicators include their microbial content, particularly *Salmonella*, *Shigella*, and *E. coli* O157:H7, as well as their PTE content.

The current study aims to evaluate the efficiency of some modern technologies used in treating contaminated soil and agricultural wastewater to obtain tomato fruits suitable for human consumption under a greenhouse scale, as well as evaluate the application of the best treatment(s) gathered from greenhouse work at the field scale.

## Materials and methods

### Greenhouse experiment

A completely randomized split-plot design experiment was conducted in three replicates at the greenhouse of the National Research Center of Cairo (Egypt) to investigate the impact of soil ecosystem and agricultural drainage water remediation on the safety of tomato fruits (*Solanum lycopersicum* L. var. *cerasiforme*). The main plots were designated for remediated and non-remediated soil ecosystems, while the subplots contained agricultural drainage water, whether remediated or non-remediated. Detailed chemical characterizations of both the contaminated soil and drainage water are provided in Tables 1 and 2, respectively.

**Table 1:** Chemical characterization of El-Rahawy soil samples for their total potentially toxic element (PTE) content (oven-dry basis)

Soil No.	Soil depth (cm)	Period of farming (years)	Previous land use	Cd	Cu	Fe	Mn	Pb	Zn	Ni	Zn equivalent parameter
				ppm							
1	0–30	80	Bean	6.40	13.00	163	6.00	16.00	180	9.50	282
	30–60			11.90	19.20	92	19.58	18.70	185	6.00	272.1

**Table 2:** Chemical characterization of El-Rahawy drain water samples

Date of water sampling	Type of water according to Doneen (1954)	EC (dS m^−1^)	pH	TSS (ppm)	Doneen parameter (Cl^−^ + 0.5 SO_4_)	Ca	Mg	Na	K	SAR
						mg/l				
River Nile	Water is very good and suitable for irrigation for all crops	0.48	7.73	307.2	1.32	2.35	0.15	2.33	0.52	1.40
El-Hode Site	Unsafe to use such water for a long time in irrigation without some precautions that should be applied	1.95	7.37	1248	15.12	3.11	4.18	11.8	0.25	1.80
El-Hadar Site		2.0	7.60	1280	16.09	3.86	3.77	10.6	0.20	1.76

Sufficient numbers of 30 cm^3^ pots were filled each with 4 kg of either remediated or non-remediated surface soil ecosystem collected from El-Rahawy Village, Giza Governorate, fertilized with recommended levels of NPK. Three tomato seedlings (*Solanum lycopersicum* L. var. *cerasiforme*) obtained from a private nursery at Giza Governorate were sown in September 2021. Irrigation water was brought from the El-Rahawy agricultural drain at Giza Governorate. All pots were eventually irrigated with either remediated or untreated agricultural drainage water to maintain proper humidity levels in the soil ecosystems during the whole experimental period.

## Remediation practices

### Premeditative amendments

To select the best treatment(s), five treatments were employed for the remediation of contaminated soils in the field experiment; these treatments are represented as follows:

1. Untreated soil irrigated with untreated agricultural drainage water CCU (control 1)
2. Untreated soil and irrigated with DHS remediated agricultural drainage water CCT (control 2)
3. Soil remediated with an equivalent mixture of bentonite, rock phosphate, and elemental sulfur, inoculated with phosphate-dissolving bacteria and *Thiobacillus* sp., and irrigated with untreated agricultural drainage water (T1) according to Kabary et al. (2021a)
4. Soil remediated with a combination of bentonite, rock phosphate, and elemental sulfur, inoculated with phosphate-dissolving bacteria and *Thiobacillus* sp., and then irrigated with remediated agricultural drainage water (T2) according to Kabary et al. (2021a)
5. Soil remediated with an equivalent mixture of dissimilar clay mineral types (ball clay, Aswan clay, bentonite, and kaolinite) with phosphate-dissolving bacteria and *Thiobacillus* sp. and irrigated with remediated agricultural drainage water (T3) according to Wahba and Zaghloul (2007), Zaghloul and Saber (2019), and Saber et al. (2016a & b and 2019).

### Isolation and cultivation of microorganisms used in the bioremediation trails

Cultures of the bacteria *Acidithiobacillus ferrooxidans* (Atlas 2005), *Acidithiobacillus thiooxidans* (Camacho et al. 2020), *Bacillus megaterium* var *phosphaticum* (Narendra and Lingayya 2019), and fungi *Trichoderma* sp. (Kumar et al. 2012) were grown in BioFlo and CelliGen fermentor/bioreactor each in its specific medium until reach 10^6^ CFU. Each microbial culture was immobilized on an appropriate mordant at the rate of 20 ml microbial culture per 100 g mordant (oven-dried soil). Sewage soils were exclusively inoculated with a single microorganism at the rate of 100 g impregnated mordant/400 g soil.

### Agricultural drainage water bioremediation and effluent quality

#### Reactor design and operation

DHS reactor was constructed and conducted according to Eleshmawiy et al. (2022) to remediate the agricultural drainage water collected from the El-Rahawy drain under continuous flowing mode. The types and concentrations of PTEs of El-Rahawy drainage water before remediation are present in Table 3, and the water quality strictures of the El-Rahway drain after DHS treatment and deduction competence are presented in Table 4.

**Table 3:** Potential toxic element (PTE) content in drainage water samples collected from El-Rahawy drain

Water samples	PTEs concentrations (ppm)				Comment
	Cd	Cu	Mn	Zn	
xSafe level	0.01	0.2	0.2	2.0	• Before application, the DHS technique urgent call to remediate El-Rahawy’s drainage water for sustainable ecosystem management • Application of DHS technique significantly purified the irrigation water
Nile water	–	0.01	–	0.01	
El-Hode site	1.0	0.45	0.93	4.14	
Water after DHS	ND	0.03	0.02	0.92	

*ND* not detected, *DHS* down-flow hanging sponge (DHS) system

**Table 4:** The water quality parameters of El-Rahway drain after DHS treatment and removal efficiency

Parameter	Unit	Raw wastewater	Characteristics after DHS treatment	%Removal efficiency	Article 51-Decree 92 of the Law of 48 in 2013
TS	mg/l	635.4	61.8	30	–
VS	mg/l	472.5	47.4	90	–
VSS	mg/l	314.1	17.1	89	–
TCOD	mg/l	616.6	22.7	95	≤ 30
SCOD	mg/l	196.3	12.8	96	–
BOD_5_	mg/l	185.2	18.1	93.46	≤ 20
DO	mg/l	1.20	5.2	–	Not less than 4
Turbidity	NTU	56.21	20.31	64.61	–
Fecal coliform	MPN	7 × 10^5^	ND*	100	1 × 10^3^
Total Coliform	MPN	6.8 × 10^7^	ND*	100	5 × 10^3^
Total count	CFU/g	93 × 10^6^	6 × 10^6^	–	–
*Pseudomonas* sp.	CFU/g	27 × 10^6^	232 × 10^5^	–	–
Phosphate-dissolving bacteria	CFU/g	1 × 10^6^	ND*	–	–
Total fungi	CFU/g	1 × 10^6^	2 × 10^5^	–	–
*Azospirillum* sp.	CFU/g	16 × 10^*5*^	28 × 10^5^	–	–
*Azotobacter* sp.	CFU/g	1 × 10^3^	15 × 10^4^	–	–
Zn	mg/l	ND*	ND*	–	2
Cu	mg/l	0.0182	0.0092	49.45	1
Ni	mg/l	0.0182	0.0092	49.45	–

*TS* total solids, *VS* volatile solids, *VSS* volatile suspended solids, *COD* chemical oxygen demand, *BOD*_*5*_ biochemical oxygen demand, *DO* dissolved oxygen, *ND* not detected

In 25-l capacity polyethylene containers, monthly samples of raw agricultural drainage water were taken from the El-Rahway drains. Investigating the assembled samples of agricultural drainage water was made on their collection day to withdraw further microbial degeneration of the prevailing contaminants. Samples were examined for different parameters according to the accepted techniques for wastewater analysis (Eaton et al. 2005). BOD, COD, TSS, dissolved oxygen (DO), pH, and turbidity were among the parameters that were examined. According to Egyptian regulations (Law 48 for 1982) (Baird et al. 2017), the obtained parameters were compared to the water quality standards.

### Field experiment

A completely randomized field experiment with three replicates was employed at the El-Hode location in El-Rahawy Village (Giza Governorate) to evaluate the best treatments gathered from the greenhouse experiment on soil ecosystem and agricultural drainage water remediation on the safety of tomato fruits under field conditions. Prior to cultivating seedlings of tomato that were obtained from a private nursery, the soil surface (0–60cm) of all experimental plots was cleaned, tilled, leveled, and split into enough number experimental plots each with an area of 12.2 m^2^ and has five rows each of 3.5 × 0.7 meter.

Seedlings of tomato were cultivated on one side of the row at a distance of 30 cm apart. All experimental plots were fertilized with recommended levels of NPK and eventually irrigated during the whole experimental period to keep a proper level of humidity in the soil ecosystem at 60% of the total water-holding capacity. The field experiment was performed in split-plot design, and the main plots encompassed remediated and non-remediated soil ecosystems, while the types of irrigation water whether remediated or untreated agricultural drainage water were laid in subplots.

Tomato plants were fertilized after 1 and 2 months of seedling in greenhouse and field experiments with the recommended levels of macro- and micronutrients represented by 150 kg ammonium sulfate/acres (20.6% N), 200 kg/acres calcium super phosphate (15.5 % P_2_O_5_), and 50 kg/acres potassium sulfate (48% K_2_O) and a mixture of micronutrients. Mature tomato fruits were collected from each treatment, and their contamination states were determined in terms of potentially toxic element (PTE) content and existing intensities of pathogenic bacteria.

The field experiment involved growing the same type of tomato used in the greenhouse one. They were treated with the best treatments taken from the greenhouse experiment, in addition to untreated soil irrigated with untreated agricultural wastewater.

### Tomato fruits safety

The safety of tomato fruits was investigated through the analysis of its potentially toxic elements’ residues (heavy metal residues) as well as the presence of pathogenic bacteria.

### Potential toxic element analyses

According to the EC (2014), tomato fruit samples were digested using a microwave (closed system). Briefly, a 1 g of homogenized sample was weighed and placed into the PTFE digestion vessels. Then, 9 ml of nitric acid (69%) and 1 ml H_2_O_2_ were added to the sample, and the tightly closed vessel was transferred to the microwave until complete digestion. The temperature-controlled program used was heating to 200 °C, holding time, and cooling to 85 °C for 15 min each. After cooling the vessels, contents were transmitted to 25-ml volumetric flasks, diluted with ultrapure water to the mark, and then ready for analysis by atomic absorption. Analysis for investigated PTEs was performed using the Atomic Absorption Spectrophotometer ICE 3500 series (Thermo) according to Abdel-Rahman et al. (2018).

### Microbiological profile of cultivated soil and tomato fruits

Ten-gram portion of each homogenate tomato sample was added in 90 ml of 0.89% (w/v) sodium chloride solution. Successively, serial tenfold dilutions were made from such homogenate tomato samples via 0.89% (w/v) physiological saline solution. The same method was replicated to estimate the intensity of total fecal bacteria and *Salmonella* sp. counts in the soil ecosystem in each trial, separately.

#### Aerobic plate counts

The total viable aerobic counts were verified according to the FDA (2002) using plate agar counts at 35 °C, for 48 h, and the plates were counted for their total population (CFU/g).

#### Coliforms intensities

*Escherichia coli* was counted according to the standard method reported by the FDA (2002) and described by El-Hadedy and El-Nour (2012), and their counts were detected by the MPN index (Bartram and Ballance 1996). A full loop of the positive tube was transferred to sterile MacConkey broth and incubated at 45.5 °C for 24 h to distinguish the fecal coliforms. EMB Agar plate was streaked from the positive tube (45.5 °C), and *Escherichia coli* appeared as purple colonies with a green metallic sheen (Gomezduarte et al. 2010).

#### *Staphylococcus* sp.

*Staphylococcus* sp. was examined according to Ollis et al. (1995), using Baird-Parker agar (Oxoid). Exactly 0.1 ml of a sample dilution was inoculated on the surface of the Baird-Parker agar plate using a sterile glass spreader. Next to incubation at 37 °C for 24 h, colonies were characterized by a grey-black color with a shiny appearance and were enumerated as *Staphylococcus* sp. The presence of *Staphylococcus aureus* was verified by the presence of coagulase-positive, catalase-positive, and gram-positive clustered cocci (APHA 1976; FDA 2002).

#### *Salmonella and Shigella* sps.

*Salmonella and Shigella* sp. detection was accomplished as given by the FDA (2002). A 25 g portion of the accurately homogenized sample was added to 225 ml of sterile buffered peptone water for pre-enrichment. After incubation at 37 °C for 24 h, 10 ml of the growth suspension was transmitted to 90 ml selenite broth enriched with 4 g l^−1^ sodium bi-selenite (Oxoid) and incubated at 37 °C for 24 h. After incubation, XLD plates were streaked from selenite broth (Gomezduarte et al. 2010). Colonies were distinguished as *Salmonella* when seen red with black centers or *Shigella* when existed reddish. The presence of *Salmonella* was validated by recording catalase-positive, urease-negative, and gram-negative short rods. Further validation was done through a triple sugar iron test. The positive isolate displayed glucose fermentation (yellow button), H_2_S positive (blackening), but negative gas production.

#### Enterobacteriaceae

Enterobacteriaceae was counted on Violet Red Bile Glucose (VRBG) agar. After incubation at 37 °C for 24 h, the typical red-violet colonies with a diameter of 0.5 mm or more, sometimes surrounded by a reddish zone, were counted (ISO 21528-2: 2017).

### Health risk assessment of tomato produced from different remediation techniques

#### The estimated daily intake (EDI)

The estimated daily intake (EDI) of potentially toxic elements detected in tomato fruits produced from different remediation treatments was calculated using the highest detected concentrations in this work. The EDI was calculated from the following formula according to Yaacob et al. (2018) and Hussain et al. (2019):

$$\mathrm{EDI}={C}_{\mathrm{m}}\times {C}_{\mathrm{f}}\times {D}_{\mathrm{f}\mathrm{ood}\kern0.5em \mathrm{intake}}/{B}_{\mathrm{average}\kern0.5em \mathrm{weight}}$$

where *C*_*m*_ is the detected metal concentration in tomato fruits (mg/g), *C*_*f*_ is the conversion factor of 0.085 used to convert fresh-weight vegetables to dry weight, *D*_food intake_ is the daily intake of vegetables for an adult and was taken as 0.345 kg/person/day, and *B*_average weight_ is the body weight taken as 70 kg for an average Egyptian adult.

#### Target hazard quotient (THQ)

Health risk associated with feeding contaminated vegetables was measured on the basis of the target hazard quotient (THQ). The THQ is the parameter which considers assessing the non-carcinogenic effects as described by Ezemonye et al. (2019) and Agoro et al. (2020). The THQ is determined by the following formula:

$$\mathrm{THQ}=\mathrm{EDI}/\mathrm{RfD}$$

where EDI is the estimated daily intake calculated before and RfD is the oral reference dose (μg/kg/ day). The RfD values were obtained from standard assumptions of USEPA risk analysis (USEPA 2000).

## Results and discussion

The results in Table 3 generally indicated that the DHS technique resulted in significantly lower concentrations of all PTEs studied compared to the safe level of PTEs in irrigation water (Maleki et al. 2013). For example, passing water through a DHS device reduced the cadmium concentration from 1 ppm to a non-detectable value. The same trend was also found in copper, manganese, and zinc; the numerical values of these pollutants reached 0.45, 0.93, and 4.14 and decreased to 0.03, 0.02, and 0.92 ppm. Although the data is not shown, the results showed that the wastewater that passed through the DHS device significantly reduced the nickel concentration to an undetectable level.

### Effect of employed remediative amendments and type of drainage water used in irrigation on potentially toxic element status in El-Rahawy cultivated soil

From the greenhouse experiment, results showed that T1 and T3 were the best treatments for minimizing the hazards of PTEs and microorganisms. Based on this conclusion, the treatments applied in the field experiment were as follows:

1. Untreated soil and irrigated with untreated agricultural drainage water CCU (control 1)
2. Untreated soil and irrigated with DHS remediated agricultural drainage water CCT (control 2)
3. Soil remediated with an equal mixture of bentonite, rock phosphate, and elemental sulfur, inoculated with phosphate-dissolving bacteria and *Thiobacillus* sp., and irrigated with untreated agricultural drainage water (T1)
4. Soil remediated with an equal mixture of different clay mineral types (ball clay, Aswan clay, bentonite, and kaolinite) with phosphate-dissolving bacteria and *Thiobacillus* sp. and irrigated with remediated agricultural drainage water (T3)

Table 5 shows that cultivation of tomato-only CCU decreased the concentration of Zn from 185 ppm in the original soil to 123 ppm, and the consistent values for other pollutants were lowered from 19.5 and 9.5 ppm to 5 and 8 ppm for Cu and Ni, respectively. Application of rock phosphate PR to the abovementioned cultivation treatment (T1) with PDB (phosphate-dissolving bacteria) and sulfur mixed with *Thiobacillus* decreased Zn concentration to 62 ppm where the supplement of bentonite T1 significantly lowered the intensity of all pollutants studied in the soil. For example, the application of T1 decreased the concentration of Ni and Cu from 9.5 and 19.5 in CCU to 4 and 3 mg/kg, respectively.

**Table 5:** Total concentrations of potentially toxic elements in the studied soils after chemical remediation treatments applied in field experiment

Remediation treatments	Zn equivalent	Ni	Cu	Zn
	**( ppm)**			
CCU	**300**	**9.5**	**19.2**	**185**
CCT	179	5	8	123
T1	62	4	3	50
T3	46	1	2	40
FAO/WHO (2001)	**Not mentioned**	**50**	**100**	**60**

Rock phosphate (RP) or soluble phosphate was formerly reported as a means of chemical treatment for many PTEs altering them into less available, insoluble forms. Different forms of phosphates such as potassium, sodium, hydrogen, and dihydrogen phosphates insolubilize divalent PTE cations resulting in the formation of insoluble orthophosphate compounds that are stable in the natural environment (Saber et al. 2012).

Results also exhibited that applying premeditative material meaningfully reduced Pb concentration by about 80% in the soil, irrespective of the treatments applied. Application of all used clay minerals (T2) exhibited variations in the percentage reduction for different pollutants. It is clear that T2 treatment decreased Cd in soils by the ranges of 54–84%, Ni more than 90%, Cu more than 82%, and Zn about 87% of total concentrations of pollutants, and it should be mentioned that the highest percentages of pollutants retention were almost observed in Cu. Clay minerals are minor particles of naturally enduring earth elements that are initially comprised of water, silica, and alumina (Kennedy 1990). PTEs combined with clay minerals make them relatively inert and less available (non-labile forms), as reported by Okoro et al. (2012). In addition, Usman et al. (2006) exemplified the effect of many natural materials, such as apetite clay minerals and waste products, as effective absorbents and potent alternatives to current remediation approaches. These approaches are stipulated by simple application techniques, either through direct mixing with agricultural soil or placement as a linear near the desired sites.

**Fig. 1:**
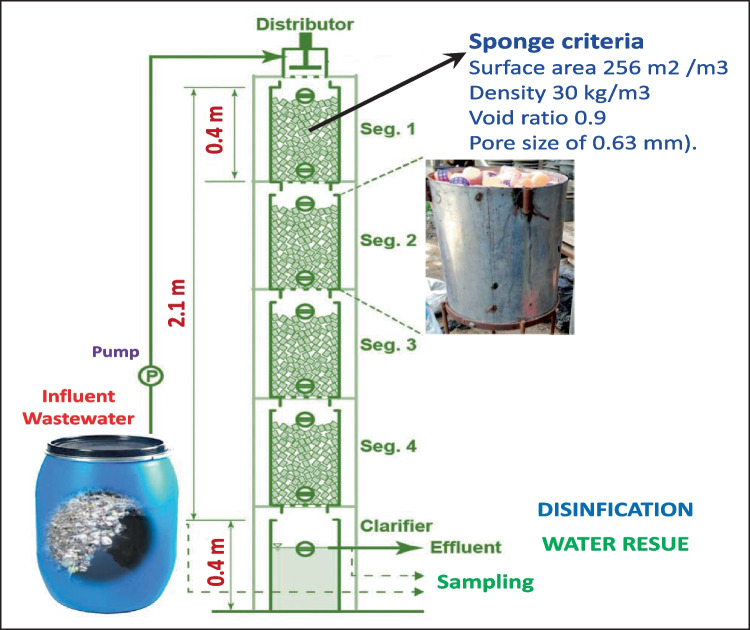
Schematic diagram of DHS reactors and sponge size

The mixture of all clay minerals with remediation treatments applied was the best treatment for reducing the inorganic pollutants hazards of the studied soils to a safe level according to WHO/FAO values. Saha et al. (2002) portrayed the effect of metal ion concentration on soil particle absorption competencies. Naturally, when metal ions are found in low concentrations, these ions bound on specific sites of soil particles; however, as the metal concentrations increase, ions lie down due to non-specific binding and a lower soil adsorption coefficient value (Kd) (Yu et al. 2002; Sastre et al. 2006). The same trend was described by Mansour et al. (2022), and they showed the effect of natural and modified clay minerals (bentonite and kaolinite) on the removal of potentially toxic elements (Cu, Zn, Ni, Cd, Cr, and As) from contaminated soil. Figure 1 represents schematic diagram of DHS reactors used in remediation of polluted drainage water. This device developed in National Research Centre (NRC) to clarify the drainage water.

### Effect of the employed remediative techniques on the safety of tomato fruits

#### Potential toxic element (heavy metal residues) concentrations in tomato fruits

**Fig. 2:**
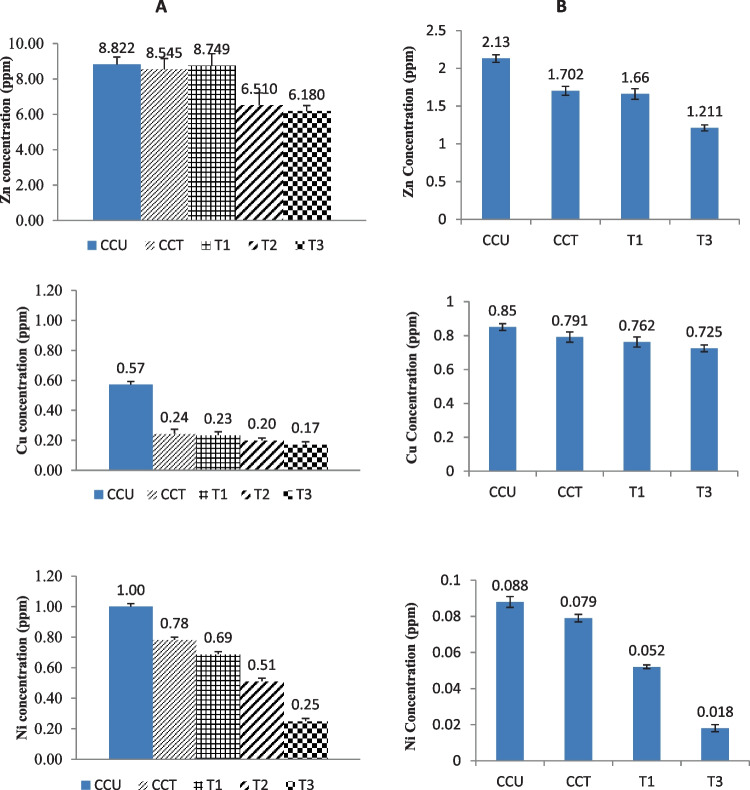
Potential toxic elements concentrations in tomato fruits grown in remediated and non-remediated soils irrigated with remediated and non-remediated agricultural drainage water at the greenhouse experiment (**A**) and field experiment (**B**)

Tomato plants are frequently eaten raw and exploited in cooking most of our food dishes, besides being an imperative export vegetable crop that sustains the national economy. Tomato fruits can absorb and accumulate a variety of potentially toxic elements in their tissues when grown in a contaminated soil ecosystem and/or irrigated with contaminated, low-quality water. Figure 2 represents the concentrations of PTEs measured in tomato fruits grown in remediated or non-remediated soil ecosystems and irrigated with remediated or non-remediated agricultural drainage water on a greenhouse scale and a field scale. It is well known that the invasion of potentially toxic elements into the human body leads to serious adverse impacts on their health, even at low concentrations, due to the absence of an effective excretion mechanism (Ghosh et al. 2012).

The PTE content detected in tomato fruits grown in the El-Rahawy soil ecosystem might cause some adverse impacts on human health, even at low concentrations (Ghosh et al. 2012). Results confirmed that Cd content in tomato fruits in control and different treatments applied were not detected either in greenhouse or field-scale experiments. This result is consistent with the objective of the research of having clean fruits suitable for export and below the maximum permissible limits (0.1 mg/kg) as set by EC (2014).

The obtained results in Fig. 2A revealed that all applied treatments decreased the uptake of PTEs in tomato fruits under the greenhouse experiment scale. Zinc (Zn) concentrations were decreased from 8.82 ppm in the CCU control treatment to 6.18 ppm in the T3; it should be mentioned that Cu and Ni concentrations gave the same trend of reduction. The concentrations of Cu and Ni were reduced from 0.57 and 1.0 ppm in CCU control treatment to 0.17 and 0.25 ppm in T3 respectively with reduction percent equal to 60% and 75 % for Cu and Ni, respectively.

Potential toxic element (PTE) concentrations in tomatoes at the field scale showed the same style of results illustrated in Fig. 2B. Simultaneously, T3 treatment showed the highest Zn, Cu, and Ni reduction from the highest control CCU treatment. The reduction percentage achieved by applying T3 treatment was 43.1%, 14.7%, and 79.5% for Zn, Cu, and Ni, respectively. The detected intensities of potentially toxic elements in all tomato samples were below the guidelines for safe levels (Chauhan and Chauhan 2014). Environmental contamination with PTEs, especially in soil ecosystems, represents a serious potential hazard to our health due to their uptake by plants and following introduction to animals and humans via the food chain. Remediation of soil ecosystems from PTEs has a major role in the production of safe food (Wang et al. 2003). Vegetables are the main edible food that presents an indispensable part of the human diet. PTEs could be absorbed by vegetables through their roots and gathered at high concentrations in the edible portions of these vegetables, regardless of whether they are detected in the soil ecosystem at low levels (Zhou et al. 2016).

Inorganic pollutants in tomato fruits were assessed by Sulaiman et al. (2019); they detected concentrations of 1.02, 0.11, 0.43, and 2.0 mg/Kg for Cd, Ni, Cu, and Zn, respectively, and this result agrees with our findings. In addition, results reported by Chowdhury et al. (2019) showed that the concentrations of PTEs accumulated in tomato fruits grown in industrial-contaminated soils were higher than those reached in the current work; this result could be related to the significant variations between the types of waters used in irrigation in their PTE contents and their electronegativity. Moreover, the lower production of vegetables grown in such soil ecosystems may be due to their higher contamination with PTEs, which react with applied fertilizers, as evidenced by the increased accumulation of heavy metals in tomato samples collected from untreated UCC plots.

Zinc is an important element regulating the immune system in the human body. Alexander et al. (2006) stated that the lack of zinc in the human diet has many devastating effects. Al-Jaboobi et al. (2014) evaluated the PTE concentration in some vegetables irrigated with wastewater in Morocco. They proved that while Cd was absent in tomato fruits, Zn, Cu, and Ni existed at significant concentrations that were relatively higher than those found in our findings. Also, Elbagermi et al. (2012) monitored the PTE content in some fruits and vegetables collected from different market sites in Libya and found that tomatoes contained 8.427, 2.245, 0.20, and 0.250 mg/kg, respectively, of Zn, Cu, Ni, and Cd which are in accordance with our findings except that of Cd. Abdel-Rahman et al. (2018) estimated PTE residues in Egyptian vegetables and compared their findings with those of others. They recommended tolerable limits for PTEs and concluded that the detected concentrations of Cd, Cu, and Ni in tomato samples were below the maximum residual limits of international legislation.

Saumel et al. (2012) endorsed that concentrations of Zn ions in tomato, potato, and other vegetable crops were lower than their concentrations in leafy vegetables. Moreover, Yang et al. (2010) exemplified that Cd buildup in vegetable plants is reduced in the following order: leafy vegetables > solanaceous vegetables > root vegetables > allimus vegetables > melon vegetable > legumes vegetables. Furthermore, Wang et al. (2021) reported similar conclusions for Pb and Cd contents in most leafy vegetables that were found above the criterion line, whereas relatively low concentrations were found in tomato, asparagus, bean, and other non-leafy vegetables.

Concentrations of PTEs accumulated in soils change with the daily change in the level of PTEs in irrigation waters, leading to the accumulation of inorganic pollutants in vegetables grown in these soils. Leblebici and Kar (2018) reported that the concentrations of heavy metals in vegetables irrigated with wastewater are significantly higher than in vegetables irrigated with tube well water, and in general, sewage effluents and wastewater are dangerous in the germ of PTEs, but industrial effluents have more adverse effects than wastewater, according to PTE uptake. Different strategies for remediation are used to reduce PTE levels in the soil to make it suitable for safe food production. Also, the study carried out by Shehata et al. (2019) showed the effect of the phytoremediation technique on the reduction of PTEs in polluted soils as well as in tomato plants grown in polluted and remediated soils and intercropped with radish and turnips, which are in agreement with our findings as the applied remediation treatments reduce the levels of PTEs in tomato.

#### Microbiological analyses of soil and tomato fruits grown in El-Rahawy as affected by employed remediative technology

The existence of enteric pathogens in the edible portion of vegetable fruits always exerts a significant threat to the consumer’s health. Most of such enteric pathogens cause infections that can lead to death (Ogundipe et al. 2012; Hassanain et al. 2021; Kabary et al. 2021b). The high microbial load of vegetables fruits mainly generates from irrigation water and soil ecosystems. Results illustrated in Table 6 illustrate total fecal and *Salmonella* sp. counts in El-Rahawy remediated and non-remediated pot soil samples in three stages of tomato pot experiments: initial, vegetative, and maturation stages. Soil ecosystem is usually rich in organic compounds, which facilitate the growth of all microorganisms including pathogenic bacterial types. The total number of fecal bacteria in El-Rahawy soil reached 8 × 10^5^ and 6 × 10^4^ viable cell oven-dry basis after frequent irrigation with raw and remediated water, respectively. Pathogenic bacterial consortia are capable to proliferate and grow on the soil organic material for extended periods even after switching the polluted irrigation water source with low organic carbon, reclaimed water. On the other hand, remediating the soil with clay minerals mixture or bentonite/sulfur/phosphate together with microbial inoculum (*Acidithiobacillus* and phosphate-dissolving bacteria) resulted in demolishing pathogenic bacterial count to 2 × 10^3^ and 3 × 10^3^ viable cell oven-dry basis after irrigation with reclaimed and drainage water at the end of maturation stage, respectively. Similarly, the *Salmonella* count was decreased in remediated soil from 7 × 10^2^ to 30 and 10 CFU (g/ml) for the soil irrigated with raw drainage and reclaimed water, respectively. Our results agreed with Saber et al. (2022) who demonstrated the role of microbial amalgamated bentonite on partial and complete removal of total coliform bacteria and *Salmonella* sp. from polluted soil groups collected from different locations in Egypt.

**Table 6:** Total fecal coliforms and *Salmonella* sp. intensities in El-Rahawy soil pot experiment (CFU/g)

Treatments	Total coliform count			*Salmonella* spp.		
	Initial	Vegetation	Maturation	Initial	Vegetation	Maturation
CCU	8 × 10^5^	4 × 10^4^	1.5 × 10^5^	7 × 10^2^	9.7 × 10^3^	6 × 10^2^
CCT	6 × 10^4^	5 × 10^4^	1.7 × 10^5^	7 × 10^2^	9 × 10^3^	4 × 10^2^
T1	7 × 10^4^	1 × 10^3^	3 × 10^3^	2 × 10^2^	40	30
T2	4 × 10^4^	5 × 10^3^	5 × 10^3^	4 × 10^2^	70	50
T3	4 × 10^4^	1 × 10^3^	2 × 10^3^	1.6 × 10^2^	20	10

CCU: untreated soil irrigated with untreated agricultural drainage water (control 1). CCT: untreated soil and irrigated with DHS remediated agricultural drainage water (control 2). T1: soil remediated with an equivalent mixture of bentonite, rock phosphate, and elemental sulfur, inoculated with phosphate-dissolving bacteria and *Thiobacillus* sp., and irrigated with untreated agricultural drainage water. T2: soil remediated with a combination of bentonite, rock phosphate, and elemental sulfur, inoculated with phosphate-dissolving bacteria and *Thiobacillus* sp., and then irrigated with remediated agricultural drainage water. T3: soil remediated with an equivalent mixture of dissimilar clay mineral types (ball clay, Aswan clay, bentonite, and kaolinite) with phosphate-dissolving bacteria and *Thiobacillus* sp. and irrigated with remediated agricultural drainage water

Table 7 presents the microbial intensities found in the tomato fruits grown in a greenhouse experiment.

**Table 7:** Microbiological analyses of tomato cultivated in El-Rahawy as affected by treatments applied (greenhouse scale)

Treatments	Aerobic plate count (CFU/g)	Total coliform count (MPN/g)	*E. coli*	*Salmonella* spp.	*Staphylococcus* count (CFU/g)	*S. aureus*	Enterobacteriaceae
CCU	2 × 10^3^ ± 0.0	Nil	−ve	−ve	1 × 10^2^ ± 0.0	−ve	Nil
CCT	6 × 10^3^ ± 0.4	Nil	−ve	−ve	nil	−ve	Nil
T1	2 × 10^2^ ± 0.4	Nil	−ve	−ve	1 × 10^2^ ± 0.0	−ve	Nil
T2	2 × 10^3^ ± 0.2	Nil	−ve	−ve	1 × 10^2^ ± 0.0	−ve	Nil
T3	9 × 10 ± 0.0	Nil	−ve	−ve	Nil	−ve	Nil

The obtained results revealed that aerobic total count ranged between 2 × 10^3^ and 9 × 10 CFU/g for CCU control treatment and T3 treatment, respectively. In concern with *Staphylococcus* count, it ranged from Nil to 1 × 10^2^ CFU/g. For all treatments applied, all samples were certainly free from *S. aureus* and all members of Enterobacteriaceae including both *E. coli* and *Salmonella* spp.

Furthermore, results from the pot experiment confirmed the results obtained from the field experiment, illustrated in Tables 8 and 9. Initially, bacterial count in cultivated, control soil irrigated with reclaimed water was less than in other control soil trials, especially in the case of total fecal bacterial count (8 × 10^6^ CFU g^−1^). Our remediation trials showed also a less pathogenic bacterial count that reached 1 × 10^4^ in soil irrigated with reclaimed water. Moreover, during vegetative and maturation stages, the bacterial count also decreased to 1 × 10^4^ CFU g^−1^ for total fecal bacteria with no detection of *Salmonella* sp., respectively. Notably, the maturation stage showed a few degrees of increase in the pathogenic count when compared with the vegetative stage. The results also indicate that *Salmonella* removal was more detectable in soil than total fecal bacteria which may be explained by more total fecal bacteria initially detected in the soil sample from the beginning of the field experiment (Table 8).

**Table 8:** Total fecal coliforms and *Salmonella* intensities in El-Rahawy soil field experiment (CFU/g)

Treatments	Total coliform count			*Salmonella* spp.		
	Initial	Vegetation	Maturation	Initial	Vegetation	Maturation
CCU	8 × 10^6^	9 × 10^6^	4 × 10^5^	4 × 10^3^	3 × 10^3^	3 × 10^2^
CCT	6 × 10^6^	8 × 10^5^	3 × 10^5^	5 × 10^3^	3 × 10^3^	1 × 10^2^
T1	9 × 10^5^	7 × 10^5^	4 × 10^4^	2 × 10^3^	4 × 10^2^	20
T3	2 × 10^5^	5 × 10^4^	1 × 10^4^	4 × 10^2^	40	0

**Table 9:** Microbiological analyses of tomato grown in El-Rahawy as affected by treatments applied (field-scale experiment)

Treatments	Bacterial plate counts	Total coliform count (MPN/g)	*E. coli*	*Salmonella*	*Staphylococcus* count (CFU/g)	*S. aureus*	Enterobacteriaceae
CCU	155 × 10^5^ ± 0.0	46 × 10	−ve	−ve	1 × 10^4^ ± 0.08	−ve	2 × 10^2^ ± 0.00
CCT	2 × 10^5^ ± 0.02	3.6	−ve	−ve	27 × 10^3^ ± 0.0	−ve	Nil
T1	54 × 10^4^ ± 4.89	Nil	−ve	−ve	51 × 10^2^ ± 0.0	−ve	4 × 10^2^ ± 0.00
T3	74 × 10^3^ ± 4.89	Nil	−ve	−ve	49 × 10^2^ ± 0.0	−ve	Nil

The results presented in Table 9 illustrate the microbiological analyses of tomato fruits affected by different bioremediation treatments cultivated in the El-Rahawy soil ecosystem (field-scale experiment). The gained results showed increased intensities of aerobic plate count, *Staphylococcus* count, and Enterobacteriaceae than that obtained in the greenhouse experiment which may be attributed to the open condition, handling, and transportation of field experiment. The T3 treatment reduced the aerobic plate count by about 3 log cycles from 155 × 10^5^ CFU/g in CCU control treatment to 74 × 10^3^ CFU/g in T3 treatment. This treatment also showed a reduction in total *Staphylococcus* and Enterobacteriaceae counts by about 2 log cycles. The total *Staphylococcus* and Enterobacteriaceae counts ranged from 49 × 10^2^ to 1 × 10^4^ CFU/g and from Nil to 2 × 10^2^ CFU/g for T3 treatment and CCU control treatment, respectively.

Aerobic plate count does not reflect food safety but is closely related to shelf life and overall product quality (Oliveira et al. 2010). It may represent the normal flora of the vegetables or attached from soil, irrigation water, and the environment during handling and transportation by workers (Ofor et al. 2009). Evaluating food eminence depends typically on perceiving the existence of indicator bacteria such as *Escherichia coli* and other coliform bacteria. However, while food quality recognition through examining other coliform bacteria is appropriate, *E. coli* is a more specific indicator of fecal pollution than the other bacterial types for the subsequent reasons: the discovery that some fecal coliforms were originally non-fecal and the development of novel superior testing methods for *E. coli* (Odonkor and Ampofo 2013). Thus, evaluating the destiny of these indicators is relevant to assess their perseverance in the environment and likely transfer to other natural water resources or to the food chain. Microbiological analysis of crop and agricultural soil was carried out to assess the presence of this fecal indicator (*Escherichia coli*) when wastewater is used for irrigation and as a parameter of water remediation efficiency (Vergine et al. 2015).

It is worth mentioning that fresh cuts of vegetables always impregnate a customary of normal flora, non-pathogenic epiphytic microorganisms. During the stages of growth, harvest, transportation, and handling of tomato fruits, they might be contaminated by other extra microorganisms especially pathogenic ones (Ogundipe et al. 2012). The consumption of such contaminated vegetables might cause a risk to consumers (Brandl 2006).

Concerning wastewater, findings of various studies have differentiated between surface microbial content and bottom microbial content regarding fecal coliforms and Enterobacteriaceae (Alam 2014). This fact is based on the sedimentation within the bottom of water that acts as a nutritional source and protects such microorganisms from UV sunlight (Mo’ataz et al. 2021), meaning that the suspended microbes included in irrigation water have a lower count because of being under stress of nutrient limitation and exposure to the UV sunlight than bottom microorganisms.

Microorganisms that are able to initiate human disease might be found in raw food harvests such as vegetables and crops. According to Hernandez-Brenes (2002), the use of irrigation water or unsanitary handling practices might be a part of the harvest surface micro-flora as incidental contaminants from the soil and its surroundings or initiated through deprived production and handling performance such as manuring with raw organic fertilizer.

As for the microbes present with vegetables, they can be disposed of or reduced in numbers by boiling acceptable levels by using several methods, including good washing with many treatments such as dilute solutions of acetic acid, chemical sterilization tablets for fresh vegetables, or washing with ozone-saturated water, which has proven to be highly efficient in getting rid of such microbes while preserving the nutritional properties of these vegetables

### Health risk assessment of tomato grown under different premeditative techniques

The estimated daily intake of PTEs detected in tomato fruits produced after different bioremediation treatments was calculated, and the obtained data is presented in Table 10. Regarding the EDI, assuming that the regular weight of an Egyptian adult is 70 kg suggests that the consumption of tomato fruits produced after remediation treatments is free from risk or that no potential risk is expected from the consumption of this tomato.

**Table 10:** Estimated daily intake (EDI) and target hazard quotient (THQ) of studied potentially toxic elements in tomato fruits

Potential toxic elements	EDI					RfDo	THQ				
	CCU	CCT	T1	T2	T3		CCU	CCT	T1	T2	T3
Zn	0.0037	0.0036	0.0037	0.0027	0.0026	0.3000	0.0123	0.0119	0.0122	0.0091	0.0086
Cu	0.0002	0.0001	0.0001	0.0001	0.0001	0.0040	0.0599	0.0255	0.0246	0.0210	0.0182
Ni	0.0004	0.0003	0.0003	0.0002	0.0001	0.0200	0.0210	0.0164	0.0144	0.0107	0.0053
Cd	ND	ND	ND	ND	ND	0.0010	ND	ND	ND	ND	ND

*EDI* estimated daily intake, *THQ* target hazard quotient, *RfDo* the oral reference dose (μg/kg/ day)

The THQ is the parameter for non-carcinogenic belongings; it is the ratio between the assessed dose of contaminants and the reference dose. The THQ values measured in this study are presented in Table 10. All measured THQ values were less than one, meaning there is no cancer risk from consuming cultivated tomatoes produced from different bioremediation treatments. This result is consistent with that reported by Yaacob et al. (2018) when evaluating toxic levels of cadmium and lead in leafy vegetables and fruits. Also, Adebiyi et al. (2020) reached the same conclusion after evaluating the health risk of PTEs in commonly consumed crayfish. However, Hussain et al. (2022) showed some THQ values over one, especially after assessing the potential risk of PTEs in vegetables irrigated with four dissimilar sources, and finally concluded that As is the metal with greater risk for non-carcinogenic outcomes than Pb and Cd.

## Conclusions

In the current work, DHS technology was used to treat agricultural wastewater used to irrigate El-Rahawy soil to reduce the risks of PTEs and produce a safe tomato crop. Biochemical compounds used in soil remediation included absorbents, low-cost stabilizers, raw materials, and microbial-fortified raw materials. Results indicated that sanitary scanning and chemical treatment techniques successfully harvest safe food crops. Furthermore, the results also showed that T3 (soil treated with mixed clay minerals (bentonite, kaolinite, ball clay, and Aswan clay), phosphate rock inoculated with phosphate-dissolving bacteria, and elemental sulfur inoculated with (*Thiobacillus* sp.)) was the best management technique for achieving a healthy and safe tomato crop. To produce safe food from contaminated soil, it is important to apply some necessary practices based on the use of treated or clean irrigation sources and applying modern technologies to minimize the risks of PTEs as well as adhering to varieties that are resistant to diseases and pathogenic microbes and then adhering to good practices during transportation and manufacturing, such as storage and handling. It is necessary to educate farmers, producers, and consumers through various awareness programs that explain the importance of adhering to good standards of agricultural practices and methods of cleaning and sterilizing vegetables, getting rid of harmful microbes, or reducing their numbers to acceptable levels.

## Data Availability

The datasets used and/or analyzed during the current study are available from the corresponding author on reasonable request.
